# How safe is growth hormone treatment during childhood?

**DOI:** 10.1186/1687-9856-2013-S1-O14

**Published:** 2013-10-03

**Authors:** Wayne Cutfield

**Affiliations:** 1 Liggins Institute and National Research Centre for Growth and Development. University of Auckland, New Zealand

## 

Since the introduction of recombinant human growth hormone (GH) in in the mid-1980s, supplies are almost limitless and predictably GH usage has escalated dramatically. There have been more than 150,000 children treated with GH worldwide with a wide variety of growth disorders.

Adverse events (AE) with all forms of drug therapy are markedly under-reported, considerably underestimating the incidence of the AE. This under-reporting occurs even when the AE is serious and has a possible or probable association with GH therapy. Careful audit of the frequency of reporting of severe AE to other drugs with a possible or probable association with treatment is only 14%. Furthermore missing data is very common and adds to the difficulty in interpreting AE.

Important conditions (adverse events) possibly linked to GH treatment are fortunately rare. There are several major limitations to accurately assessing the prevalence and risk of rare adverse events. Firstly, extremely large datasets are required found in large databases such KIGS and NCGS each with >50,000 enrolled patients. Secondly, accurate risk assessment of the possible adverse event in an untreated population that matches the GH treated population has rarely been determined.

Important potential adverse events that have received considerable attention will be addressed. These include; tumour recurrence, new malignancies including leukaemia, diabetes mellitus, benign intracranial hypertension and slipped upper femoral epiphyses. The physiological rationale and the risk of these conditions during GH treatment will be addressed.

Serum IGF-I monitoring to prevent elevated levels that may further increase the risk of adverse events is recommended. The potential adverse consequences of sustained elevated serum IGF-I levels will be discussed. In addition, monitoring IGF-I helps to monitor compliance of GH therapy in children with GH deficiency.

